# Taxonomic studies on *Amomum* Roxburgh s.l. (Zingiberaceae) in Myanmar II: one new species and five new records for the flora of Myanmar

**DOI:** 10.3897/phytokeys.138.38736

**Published:** 2020-01-10

**Authors:** Hong-Bo Ding, Bin Yang, Mya Bhone Maw, Pyae Pyae Win, Yun-Hong Tan

**Affiliations:** 1 Southeast Asia Biodiversity Research Institute, Chinese Academy of Sciences & Center for Integrative Conservation, Xishuangbanna Tropical Botanical Garden, Chinese Academy of Sciences, Menglun, Mengla, Yunnan 666303, China Southeast Asia Biodiversity Research Institute, Chinese Academy of Sciences & Center for Integrative Conservation Yunnan China; 2 Center of Conservation Biology, Core Botanical Gardens, Chinese Academy of Sciences, Menglun, Mengla,Yunnan 666303, China Core Botanical Gardens, Chinese Academy of Sciences Yunnan China; 3 Forest Research Institute, Forest Department, Ministry of Environmental Conservation and Forestry, Yezin, Nay Pyi Taw 05282, Myanmar Ministry of Environmental Conservation and Forestry Nay Pyi Taw Myanmar

**Keywords:** Kachin State, *
Lanxangia
*, *
Meistera
*, Putao District, Sangaing Region

## Abstract

In the course of a study of *Amomum* s.l. (Zingiberaceae) in Myanmar, *Amomum schistocalyx* Y.H. Tan & H.B. Ding, from Htamanti Wildlife Sanctuary, Sangaing Region of Northern Myanmar is described and illustrated as new to science here. Five taxa: *A. yingjiangense* S.Q. Tong & Y.M. Xia, *A. carnosum* V.P. Thomas & M. Sabu, *A. tibeticum* (T.L. Wu & S.J. Chen) X.E. Ye, L. Bai & N.H. Xia, *Lanxangia scarlatina* (H.T. Tsai & P.S. Chen) M.F. Newman & Škorničk, and *Meistera yunnanensis* (S.Q. Tong) Škorničk. & M.F. Newman, are newly recorded from Myanmar. The photographic illustrations, the distributions, and voucher specimens for each species are provided.

## Introduction

*Amomum* s.l. (Roxburgh 1820: 75) is the second largest genus in the family Zingiberaceae with about 150–180 species (Xia et al. 2004; Thomas et al. 2016). It is distributed from the Himalayas throughout Southeast Asia to northern Australia and extends into the central Pacific (Tripathi and Prakash 1999; Xia et al. 2004; Kaewsri and Paisooksantivatana 2007), the centre of endemism being the forests of Southeast Asia (Droop and Newman 2014). Based on molecular phylogenetic analyses by De Boer et al. (2018), *Amomum* s.l. are now categorised into seven monophyletic genera, namely *Amomum* s.s., *Conamomum*Ridley (1899: 121), *Meistera*Giseke (1792: 205), *Wurfbainia*Giseke (1792: 206), *Epiamomum* A.D. Poulsen & Škorničková (De Boer et al. 2018: 22), *Lanxangia* M.F. Newman & Škorničková (De Boer et al. 2018: 23) and *Sundamomum* A.D. Poulsen & M.F. Newman (De Boer et al. 2018: 27).

According to this new treatment, the Myanmar species previously classified in *Amomum* s.l. are now categorised into three genera, namely *Amomum* s.s. (7 species: *A. dealbatum* Roxb., *A. robertsonii* Craib, *A. sericeum* Roxb., *A. subulatum* Roxb., *A. erythranthum* Y.H. Tan & H.B. Ding, *A. ampliflorum* Y.H. Tan & H.B. Ding, *A. pauciflorum* Baker), *Meistera* (2 species: *M. aculeata* (Roxb.) Škorničk. & M.F. Newman, *M. koenigii* (J.F.Gmel.) Škorničk. & M.F. Newman) and *Wurfbainia* (4 species: *W. aromatica* (Roxb.) Škorničk. & A.D. Poulsen, *W. gramine*a (Wall. ex Baker) Škorničk. & A.D. Poulsen, *W. villosa* (Lour.) Škorničk. & A.D.Poulsen, *W. microcarpa* (C.F. Liang & D. Fang) Škorničk. & A.D. Poulsen) (Kress et al. 2003; De Boer et al. 2018; Ding et al. 2019). In the course of a study of *Amomum* s.l. (Zingiberaceae) in Myanmar, one new species, *A. schistocalyx* Y.H. Tan & H.B. Ding, is described and illustrated as new to science here. Five taxa: *A. yingjiangense* S.Q. Tong & Y.M. Xia, *A. carnosum* V.P. Thomas & M. Sabu, *A. tibeticum* (T.L. Wu & S.J. Chen) X.E. Ye, L. Bai & N.H. Xia, *L. scarlatina* (H.T. Tsai & P.S. Chen) M.F. Newman & Škorničk, and *M. yunnanensis* (S.Q. Tong) Škorničk. & M.F. Newman, are newly recorded from Myanmar here. As a result, the total number of *Amomum* s.l. recorded in Myanmar is presently raised to 19. The number of species occurring in Myanmar is still too small. Further extensive fieldwork would reveal much more species diversity of Myanmar *Amomum* s.l.

## Taxonomic treatment

### Amomum schistocalyx

Taxon classificationPlantaeZingiberalesZingiberaceae

Y.H.Tan & H.B.Ding sp. nov.

B965CE5D-2C44-55A5-95BA-FC97C011C98C

urn:lsid:ipni.org:names:77204200-1

Fig. 1

#### Diagnosis.

*Amomum schistocalyx* Y.H. Tan & H.B. Ding is similar to *A. putrescens* D. Fang (1978: 51) in having lax inflorescence, similar yellow flowers, non-tubular bracteoles and green fruits, but can be distinguished by its leaves abaxially densely appressed silvery pubescent (vs. glabrous leaves), 2-cleft ligule (vs. entire ligule), non-tubular calyx (vs. tubular calyx), epigynous glands 2 mm (vs. 5 mm).

#### Type.

Myanmar. Sangaing Region, Hkamti District, near Htamanti village. 95°22'40.42"E, 25°22'32.40"N; 135 m elev., 3 June 2019, *Y.H. Tan & H.B. Ding M5785* (holotype: HITBC!; isotypes: RAF!).

#### Description.

Clump-forming herb, 1.0–2.0 m tall. Pseudostem with 2–7 leaves per pseudostem, swollen at base, greenish yellow or brownish yellow; ligule ovate, 2-lobed to middle or bottom, 2.0–4.0 cm long, yellowish brown, membranous, fragile, pubescent, apex acute; petiole 2.0–7.0 cm; leaf blade adaxially green, abaxially silvery, elliptic to oblong-lanceolate, 17–50 × 6–10 cm, adaxially glabrous, abaxially densely appressed silvery pubescent, base attenuate, apex caudate. Inflorescence radical, 5.0–18.0 cm, arising from the rhizome, one inflorescences per pseudostem, peduncle 4.0–10.0 cm, reddish-brown, flowering part obpyramidal, 3.6–5.5 × 3.5–4.5 cm, lax with rachis visible between bracts, sterile bracts ovate, 1.6–2.7 × 1.5–2.0, yellowish brown, membranous; fertile bracts (bracteoles), non-tubular, lanceolate, 1.5–2.5 × 0.3–0.5 cm, yellowish brown, membranous, striate then soon rotting, subtending a single flower. Flowers 3.2–4.0 cm, yellow. Calyx non-tubular, split to bottom (even when flower budding), 1.0–1.5 × 0.3–0.6 cm, apex 3-toothed, membranous, yellowish brown, pubescent. Floral tube 1.0–1.2 cm long, ca. 0.4 cm wide at mouth, pale yellow, glabrous; dorsal corolla lobe oblong or ovate, 1.5–1.7 × 0.5–0.7 cm, brownish yellow and reddish towards apex, apex cucullate with a 1–2 mm long cusp, margin ciliate; lateral corolla lobes oblong, 1.5–1.7 × 0.4–0.5 cm, white towards base and brownish yellow at apex, apex rounded, margin ciliate. Labellum spreading, with red at claw, dull brownish yellow at middle and margin, obovate, entire, 1.9–2.3 × 1.0–1.2 cm, margin crisped, prominently veined, dentate, glabrous, adaxial surface pilose at base. Lateral staminodes lanceolate, adaxial surface red towards base and brownish yellow at apex, abaxial surface brownish yellow, 3–4 mm. Stamen 1.2–1.7 cm long, filament 0.4–0.7 × 0.2–0.3 cm, red at base, brownish yellow at apex, pubescent, connective pubescent, brownish yellow, anther thecae oblong, 0.7–0.9 cm long, creamy-white, dehiscing throughout their length, anther crest semi-lunar or inconspicuous trilobed, ca. 7 × 2 mm, brownish yellow, membranous, margin dentate. Epigynous glands two, oblate, ca. 2 × 1 mm, creamy, glabrous. Ovary ellipsoid, ca. 6 × 3 mm, villous; style ca. 2.7 cm long, puberulous; stigma cup-shaped, white, mouth ciliate. Infructescence up to 40 cm long, usually with 1–7 fruits reaching maturity. Capsule ovoid, 3.0–3.5 × 2.7–3.0 cm, 9-winged, green, at apex with persistent pubescent calyx, wings straight, stalk 5–7 mm.

**Figure 1.**
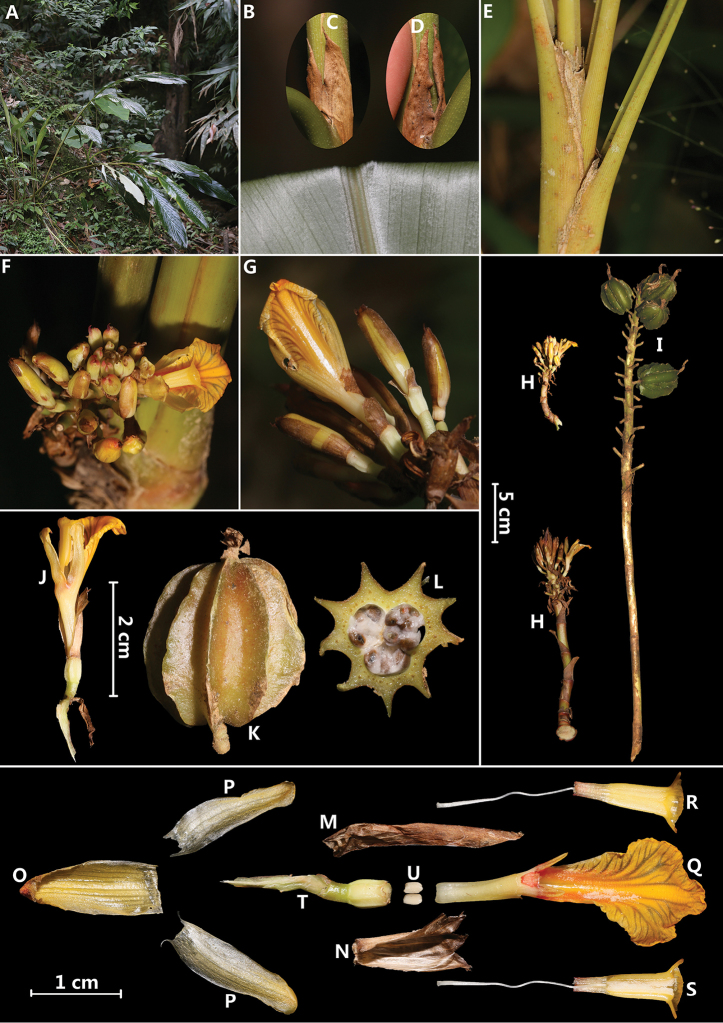
*Amomum schistocalyx* Y.H.Tan & H.B.Ding, sp. nov. **A** habit **B** leaf blade abaxially **C, D** ligule **E** pseudostem **F** flowers (front view) **G** flowers (back view) and showing non-tubular calyx (even when flower budding) **H** inflorescence **I** infructescence **J** single flower **K** single fruit **L** cross section of fruit **M** bracteole **N** calyx **O** dorsal corolla lobe **P** lateral corolla lobes **Q** labellum with floral tube and lateral staminodes **R** stamen with stigma and style (back view) **S** stamen with stigma and style (front view) **T** ovary with pedicel **U** epigynous glands. Photographed by H.B. Ding.

#### Phenology.

Flowering maybe in April–June and fruiting in May–July.

#### Distribution.

Known only from the type locality, beside the Chindwin River, Hkamti District, Sangaing Region, Myanmar.

#### Ecology.

On the riverside at an elevation of 100–150 m in tropical forest.

#### Etymology.

The specific epithet ‘*schistocalyx*’ refers to the non-tubular, dehiscent calyx.

#### Conservation status.

LC. This species is locally common. Populations in Htamanti Wildlife Sanctuary are well protected. The populations near Htamanti village are not in a protected area but the populations are robust. So we treat this species as of Least Concern (IUCN 2017).

#### Affinities.

*Amomum schistocalyx* Y.H. Tan & H.B. Ding shares similar characters with *A. putrescens* but differs in many characters, such as leaves (abaxially densely appressed silvery pubescent vs. abaxially glabrous, respectively), ligule (2-cleft vs. entire, respectively), calyx (non-tubular vs. tubular, respectively), labellum (red at claw, dull brownish yellow at middle and margin vs. almost pure yellow, respectively), lateral staminodes (lanceolate, adaxial surface red towards base and brownish yellow at apex, abaxial surface brownish yellow vs. tooth like, red, respectively) and epigynous glands (2 mm vs. 5 mm, respectively).

#### Additional specimens examined (paratypes).

Myanmar. Sangaing Region, Hkamti District, Htamanti Wildlife Sanctuary, near Nam E Zu, Camp 1. 95°28'20.56"E, 25°32'13.42"N, 141 m elev., 20 May 2019, fruiting, *B. Yang*, *H.B. Ding & X.D. Zeng M5158* (HITBC!; RAF!); Sangaing Region, Hkamti District, Htamanti Wildlife Sanctuary, near Nam E Zu, Camp 2. 95°31'34.08"E, 25°30'40.15"N, 143 m elev., 25 May 2019, fruiting, *Y.H. Tan, M. Deng, B. Yang, H.B. Ding & X.D. Zeng M5374* (HITBC!; RAF!).

##### New records for Myanmar

### Amomum yingjiangense

Taxon classificationPlantaeZingiberalesZingiberaceae

S.Q. Tong & Y.M. Xia

4AD199C5-05CF-5A7C-8A8B-CF9C67EBF8A1

Fig. 2

Amomum yingjiangense S.Q. Tong & Y.M. Xia in Acta Bot. Yunnan. 10(2): 210. 1988; S.Q. Tong in C.Y. Wu (ed.), Fl. Yunnan. 8: 622. 1997; T.L. Wu & K. Larsen in C.Y. Wu & P.H. Raven (eds), Fl. China 24: 349. 2000; De Boer et al. in Taxon 67(1): 20. 2018. Type: China, Yunnan Province, Dehong Dai and Jingpo Autonomous Prefecture, Yingjiang County, Xima Town, Huoshigou Village, 1740 m elev., 8 August 1983, *S.Q. Tong & C.J. Liao 24870* (holotype: HITBC081571!)

#### Description.

Clump-forming herb, 1–1.5 m tall, 3–7 pseudostems per clump. Pseudostem with 2–7 leaves per pseudostem, swollen and brownish yellow at base, greenish yellow towards apex; ligule ovate, entire, apex rounded, sometimes truncate or praemorse because of fragile, 5–13 mm long, densely brownish pubescent; petiole absent to 4 cm, densely brownish pubescent; leaf blade adaxially dark green, abaxially green, elliptic to narrowly elliptic, 35–65 × 5–11 cm, adaxially pubescent, abaxially densely brownish pubescent, base cuneate or attenuate, apex caudate. Inflorescence radical, 10–17 cm, arising from the rhizome, 1 inflorescences per pseudostem, peduncle short, 2–4 cm, reddish-brown, flowering part obovoid, ca. 11 × 10 cm, sterile bracts ovate, 2.5–5.5 × 2.0–4.5, reddish brown, carneous, fragile; bracteoles, non-tubular, lanceolate, 3.0–3.5 × 0.5–0.8 cm, reddish brown, carneous, fragile, subtending 1–2 flowers. Flowers 5–6 cm, white or pinkish orange. Calyx tubular, 2.3–3.0 × 0.5–0.9 cm, apex 3-toothed, membranous, white or pinkish orange, pubescent. Floral tube 2.3–2.7 cm long, ca. 0.5 cm wide at mouth, white or pinkish orange, pubescent; dorsal corolla lobe oblong, 2.2–2.5 × 0.8–1.0 cm, white or pinkish orange, hooded at apex, obtuse, ciliate; lateral corolla lobes oblong, 2.0–2.5 × 0.5–0.8 cm, white or pinkish orange, apex rounded, ciliate. Labellum spreading, obovate, white or pinkish orange with reddish stripe (by reddish dots) radiating towards apex, 2.5–3.0 × 1.5–2.0 cm, apex trilobed or inconspicuous trilobed, margin crisped or dentate, prominently veined, adaxial surface pilose at base. Lateral staminodes ovate, sometimes dentate, adaxial surface reddish, abaxial surface white or pinkish orange, 5–7 mm. Stamen 2.0–2.3 cm long, filament ca. 8 × 3 mm, white or pinkish orange, pubescent, connective pubescent, white or pinkish orange, anther thecae oblong, ca. 1.0 cm long, brownish yellow, dehiscing throughout their length, anther crest reniform or inconspicuous trilobed, ca. 7 × 3 mm, apex crenate or entire, white or pinkish orange, membranous. Epigynous glands 2, cylindrical, ca. 4 × 1 mm, creamy, glabrous. Ovary ellipsoid, ca. 5 × 3 mm, reddish brown, glabrous; style ca. 5.0 cm long, white, glabrous; stigma cup-shaped, reddish brown, mouth ciliate. Capsule not seen.

**Figure 2.**
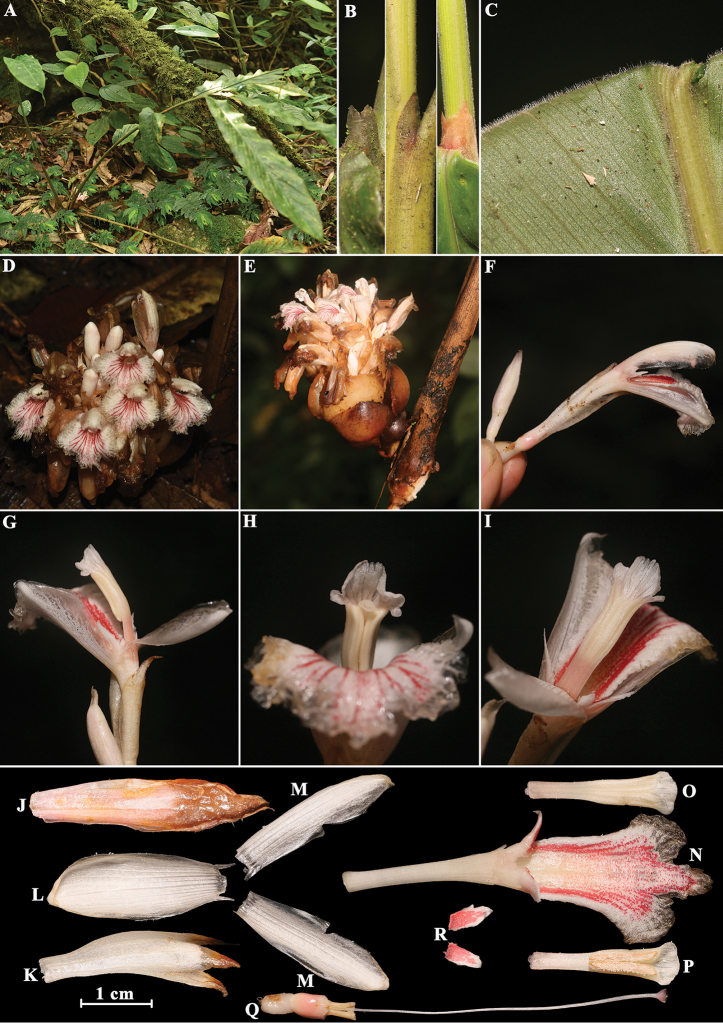
*Amomum yingjiangense* S.Q. Tong & Y.M. Xia **A** habit **B** ligule **C** leaf blade abaxially **D** inflorescence (front view) **E** inflorescence (side view) **F** flower (side view) **G** flower showing lateral staminodes **H** single flower showing anther crest (front view) **I** single flower showing stamen (back view) **J** bracteole **K** calyx **L** dorsal corolla lobe **M** lateral corolla lobes **N** labellum with floral tube and lateral staminodes **O** stamen (back view) **P** stamen (front view) **Q** ovary with epigynous glands, style and stigma **R** lateral staminodes. Photographed by H.B. Ding.

#### Specimens examined.

Myanmar, Kachin state, Putao district, Pannandin, top of mountain, understory herbs in tropical rain forest, 27°42'15.06"N, 97°52'57.88"E, 1769 m elev., flowering, 9 June 2018, *Myanmar Exped. M4262* (HITBC!; RAF!).

#### Distribution.

China, Myanmar.

#### Note.

This species was originally described by Tong and Xia (1988) from fruiting material only and recorded as endemic to China (Tong 1997, Wu and Larsen 2000). In Ye (2018), he re-described the species with flowering material from the type locality. Here, we also provide a description from Myanmar.

### Amomum carnosum

Taxon classificationPlantaeZingiberalesZingiberaceae

V.P. Thomas & M. Sabu

E2D72419-63A1-54C0-B1B6-8C4E1A5740A1

Fig. 3

Amomum carnosum V.P. Thomas & M. Sabu in Kew Bull. 67: 549. 2012; De Boer et al. in Taxon 67(1): 19. 2018. Type: India, Nagaland, Tuensang Distr., Noklak, 20 May 2007, *Thomas & Muhammed Nissar 103698* (holotype: CALI).

#### Specimens examined.

MYANMAR, Kachin state, Putao district, Upper Shankhaung to Wasandum, understory herbs in tropical rain forest, 27°27'15"N, 97°14'50"E, 1007 m elev., 17 June 2018, *Myanmar Exped. M4626* (HITBC!; RAF!).

#### Distribution.

India, Myanmar.

#### Note.

*Amomum carnosum* was first described by Thomas et al. (2012) and recorded as endemic to Nagaland, India. The species is similar to *A. maximum* (Roxburgh 1810: 344) in having a bifid membranous ligule, white flowers, but differs in its diffuse, low stoloniferous herb, 30–45 cm tall, leaves 2–3 per shoot, 3-lobed labellum, lamina elliptic and glabrous. In our collection from Myanmar, *Myanmar Exped. M4626* matches well with Thomas’s type in its morphological structure (Thomas et al. 2012). However, it differs in the slightly floral structure and having non-3-lobed labellum. Considering the floral structure of *Amomum* s.l. is easy to tear and corrupt in the rainy season, we consider that the accurate description of *Amomum carnosum* requires further observation and collection of field individuals.

**Figure 3.**
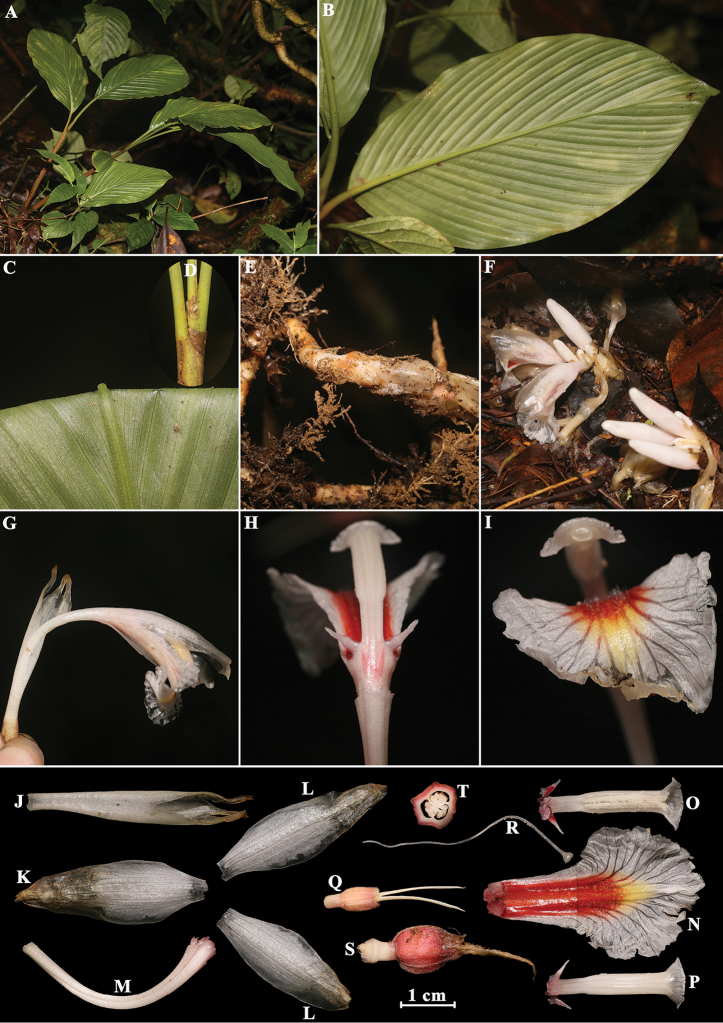
*Amomum carnosum* V.P. Thomas & M. Sabu **A** habit **B** single leaf (back view) **C** leaf blade abaxially **D** ligule **E** rhizome **F** basal part of plant showing inflorescences **G** single flower (side view) **H** single flower (back view) **I** single flower (front view) **J** calyx **K** dorsal corolla lobe **L** lateral corolla lobes **M** floral tube **N** labellum **O** stamen with lateral staminodes (front view) **P** stamen with lateral staminodes (back view) **Q** ovary with epigynous glands **R** style and stigma **S** young fruit with peduncle **T** cross section of fruit. Photographed by H.B. Ding.

### Amomum tibeticum

Taxon classificationPlantaeZingiberalesZingiberaceae

(T.L. Wu & S.J. Chen) X.E. Ye, L. Bai & N.H. Xia

6DCEE892-E354-530C-9163-B7BD2A8D048C

Fig. 4

Amomum tibeticum (T.L. Wu & S.J. Chen) X.E. Ye, L. Bai & N.H. Xia in Ye et al. Plant Syst. Evol. 304(9): 1174. 2018; –Hornstedtia tibetica T.L. Wu & S.J. Chen in Acta Phytotax. Sin. 16(3): 39. 1978; T.L. Wu & S.J. Chen, in T.L. Wu (ed.) Fl. Reipubl. Popularis Sin. 16(2): 136. 1981; T.L. Wu & K. Larsen, in C.Y. Wu & P.H. Raven (eds) Fl. China. 24: 358. 2000; –Hornstedtia arunachalensis S. Tripathi & V. Prakash, Nordic J. Bot. 19: 329. 1999. Lectotype (designated by Ye et al. 2018, pg. 1174): China. Xizang Province: Medog County, Beibeng Township (previously District), 810 m elev., 11 August 1974, *Qingzang expedition 74-1913* (PE00075268).

#### Specimens examined.

Myanmar, Kachin state, Putao district, Upper Shankhaung to Wasandum, understory herbs in tropical rain forest, 27°27'15"N, 97°14'50"E, 992 m elev., 17 June 2018, *Myanmar Exped. M4630* (HITBC!; RAF!); Kachin state, Putao district, Shinsanku, 27°39'56.90"N, 97°53'28.81"E, 990 m elev., 10 June 2018, *Myanmar Exped. M4288* (HITBC!; RAF!); Kachin state, Putao district, Naung Maung township, Khasanku village, 27°39'35"N, 97°37'21"E, 1024 m elev., 13 June 2018, *Myanmar Exped. M4457* (HITBC!; RAF!).

**Figure 4.**
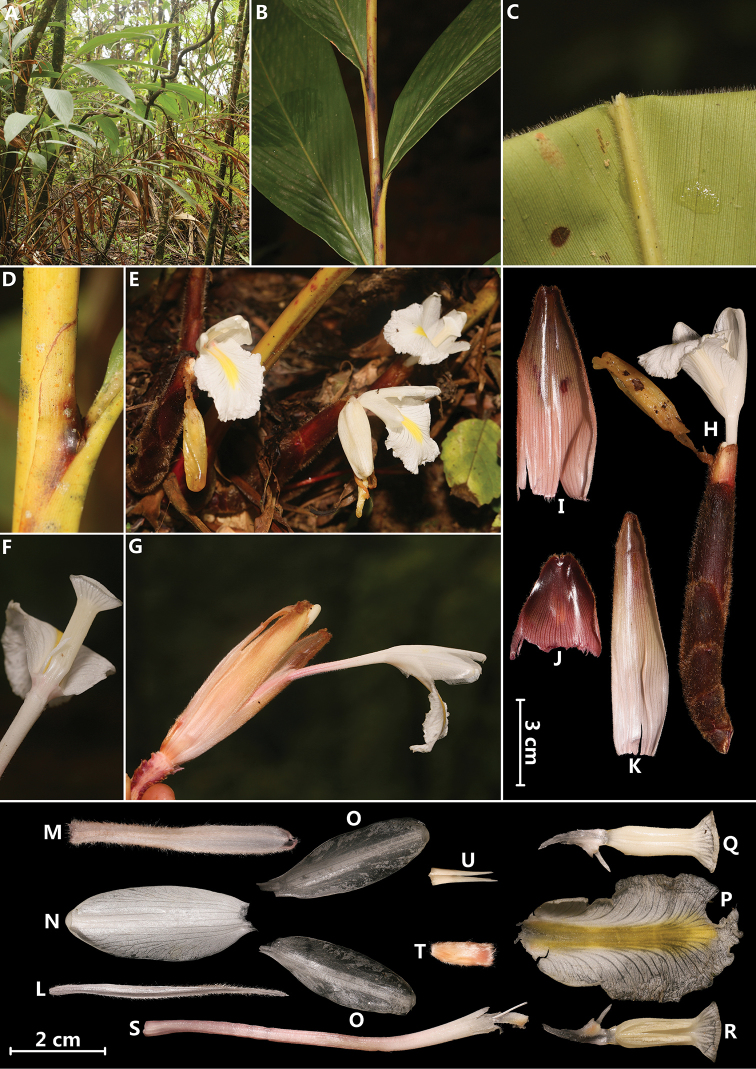
*Amomum tibeticum* (T.L. Wu & S.J. Chen) X.E. Ye, L. Bai & N.H. Xia **A** habit **B** pseudostem **C** leaf blade abaxially **D** ligule **E** inflorescences (front view) **F** single flower showing stamen (back view) **G** inflorescence (inner view) **H** inflorescence (side view) **I, J** sterile bracts **K** bract **L** bracteole **M** calyx **N** dorsal corolla lobe **O** lateral corolla lobes **P** labellum **Q** stamen with lateral staminode (back view) **R** stamen with lateral staminode (front view) **S** floral tube with style **T** ovary **U** epigynous glands. Photographed by H.B. Ding.

#### Distribution.

China, India (Ye et al. 2018), Myanmar.

#### Note.

This was originally described by Wu and Chen (1978) based on a collection from Medog County, Xizang (Tibet), China. It was placed in the genus *Hornstedtia* Retz., presumably due to the radical fusiform inflorescences covered with rigid involucral bracts enclosing oblong and smooth fruits, and pointed out that it was close to *H. affinis* Ridl. (Wu and Chen 1981). But on the basis of morphological study of flowering material originating at the type locality, the species is a member of *Amomum* s.s. (Ye et al. 2018).

### Lanxangia scarlatina

Taxon classificationPlantaeZingiberalesZingiberaceae

(H.T. Tsai & P.S. Chen) M.F. Newman & Škorničk

8116083F-8E89-5274-8B02-E53DCF097A33

Fig. 5

Lanxangia scarlatina (H.T. Tsai & P.S. Chen) M.F. Newman & Škorničk in De Boer et al. Taxon 67(1): 24. 2018; –Amomum scarlatinum H.T. Tsai & P.S. Chen in Acta Phytotax. Sin. 17(4): 90 1979; H.T. Tsai & P.S. Chen in T.L. Wu (ed.), Fl. Reipubl. Popularis Sin. 16(2): 121. 1981; S.Q. Tong in C.Y. Wu (ed.), Fl. Yunnan. 8: 639. 1997; T.L. Wu & K. Larsen in C.Y. Wu & P.H. Raven (eds), Fl. China 24: 350. 2000. Type: China, Yunnan Province, Xishuangbanna Dai Autonomous Prefecture, Jinghong City, Dadugang Township, Guanping forestry farm, 900 m elev., *J.H. Zhang 18445* (holotype: HITBC048529!).

#### Specimens examined.

Myanmar, Kachin state, Putao district, around Gathu village, understory herbs in tropical rain forest, 27°28'17.39"N, 97°57'06.58"E, 575 m elev., 1 June 2018, *Myanmar Exped. M3897* (HITBC!; RAF!); Kachin state, Putao district, from Gathu village to Tongwang Cave, understory herbs in tropical rain forest, 27°28'14.58"N, 97°57'01.01"E, 581 m elev., 3 June 2018, *Myanmar Exped. M3995* (HITBC!; RAF!).

**Figure 5.**
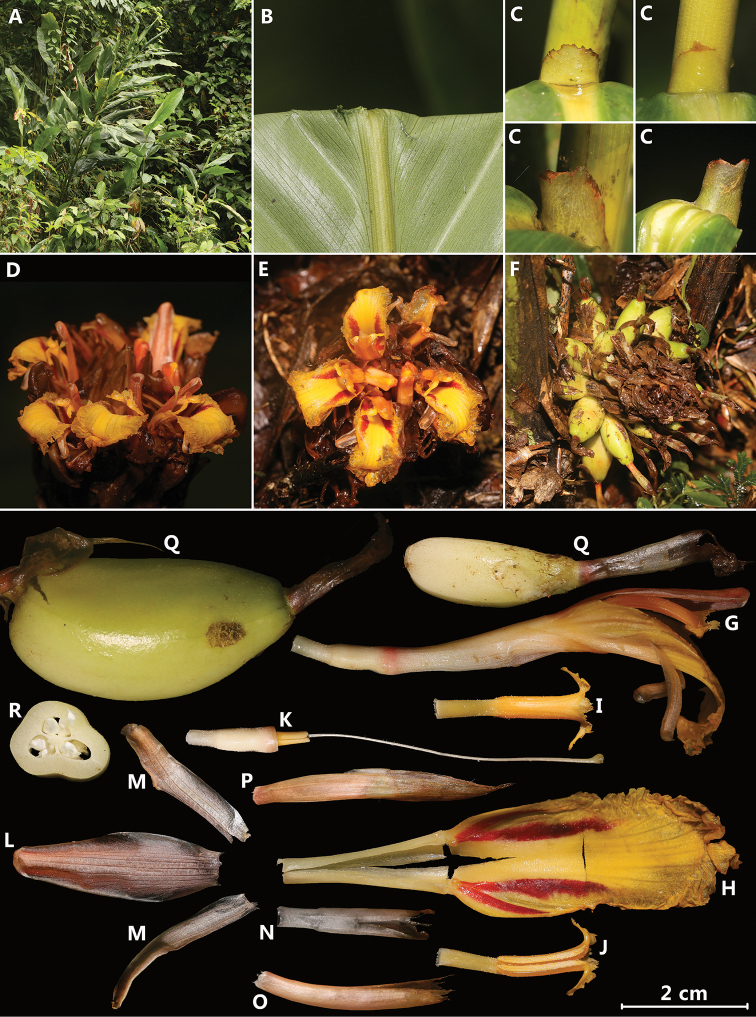
*Lanxangia scarlatina* (H.T. Tsai & P.S. Chen) M.F. Newman & Škorničk **A** habit **B** leaf blade abaxially **C** ligule **D** inflorescence (side view) **E** inflorescence (front view) **F** infructescences **G** single flower **H** labellum with floral tube **I** stamen (back view) **J** stamen (front view) **K** ovary with epigynous glands, style and stigma **L** dorsal corolla lobe **M** lateral corolla lobes **N** calyx **O** bracteole **P** bract **Q** single fruit **R** cross section of fruit. Photographed by H.B. Ding.

#### Distribution.

China, Myanmar.

#### Note.

This was originally described by Tsai and Chen (1979) based on a collection from Dadugang Township, Jinghong City, Yunnan Province, China. It was placed in the genus *Amomum* s.l., but on the basis of morphological study, the species is a member of *Lanxangia* (De Boer et al. 2018). It is a new generic record for the country.

### Meistera yunnanensis

Taxon classificationPlantaeZingiberalesZingiberaceae

(S.Q. Tong) Škorničk. & M.F. Newman

4924C075-EF33-5529-82C9-9B4338642CF7

Fig. 6

Meistera yunnanensis (S.Q. Tong) Škorničk. & M.F. Newman in De Boer et al. Taxon 67(1): 27. 2018; –Amomum yunnanense S.Q. Tong in Acta Bot. Yunnan. 12(2): 151. 1990; S.Q. Tong in C.Y. Wu (ed.), Fl. Yunnan. 8: 632. 1997; T.L. Wu & K. Larsen in C.Y. Wu & P.H. Raven (eds), Fl. China 24: 353. 2000. Type: China, Yunnan Province, Dehong Dai and Jingpo Autonomous Prefecture, Ruili county-level City, Mengxiu Township, Guangren, 1200 m elev., 25 July 1983, *S.Q. Tong & C.J. Liao 24832* (holotype: KUN1219275).

#### Specimens examined.

Myanmar, Sangaing Region, Hkamti District, Htamanti Wildlife Sanctuary, near Nam E Zu Camp 2, 25°30'05.35"N, 95°32'41.50"E, 193 m elev., 27 May 2019, *Myanmar Exped. M5515* (HITBC!; RAF!); Sangaing Region, Hkamti District, Homalin Township, just outside Htamanthi Wildlife Sanctuary, Nam Sa Bi Village Management Area, 25°18'53.50"N, 95°21'08.40"E, 216 m elev., 27 September 2016, *Kate et al. 1631* (NY02654996!).

**Figure 6.**
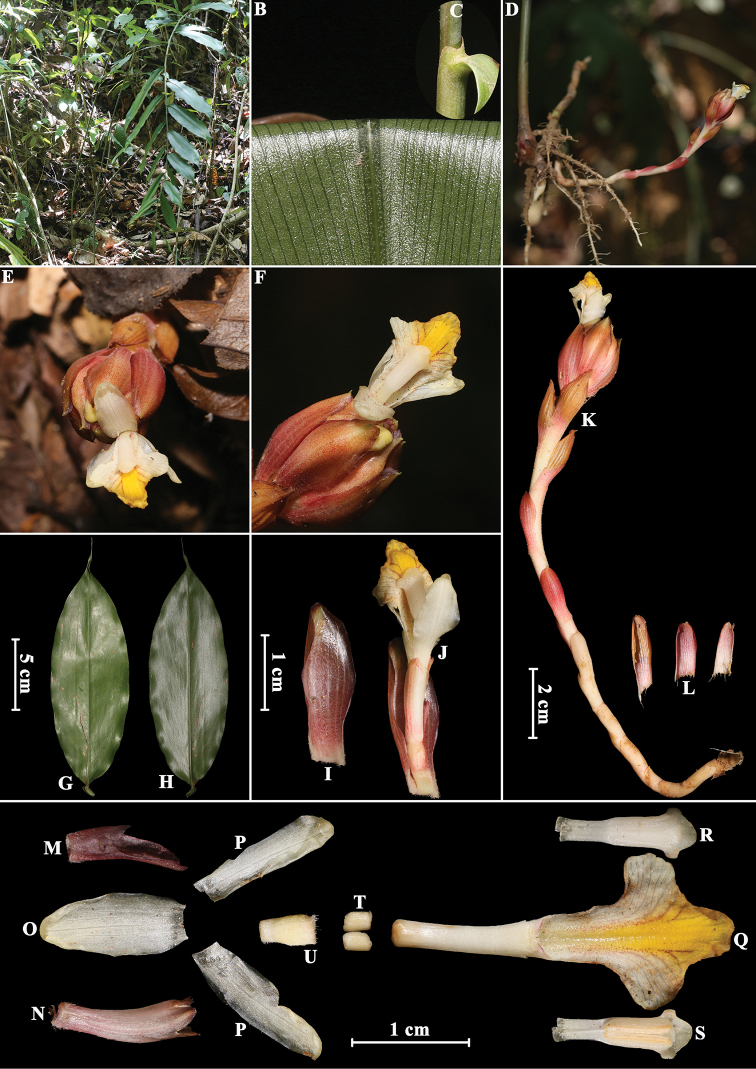
*Meistera yunnanensis* (S.Q. Tong) Škorničk. & M.F. Newman **A** habit **B** leaf blade abaxially **C** ligule **D** basal part of plant showing inflorescences **E** inflorescence **F** single flower (front view) **G** single leaf (front view) **H** single leaf (back view) **I** bract **J** single flower (side view) **K** inflorescence (side view) **L** sterile bracts **M** bracteole **N** calyx **O** dorsal corolla lobe **P** lateral corolla lobes **Q** labellum with floral tube **R** stamen (back view) **S** stamen (front view) **T** epigynous glands **U** ovary. Photographed by H.B. Ding.

#### Distribution.

China, India, Myanmar.

#### Note.

This was originally described Tong (1990) based on a collection from Dehong Dai and Jingpo Autonomous Prefecture, Yunnan Province, China. It was placed in the genus *Amomum* s.l, but on the basis of morphological study, the species is a member of *Meistera* (De Boer et al. 2018).

## Supplementary Material

XML Treatment for Amomum schistocalyx

XML Treatment for Amomum yingjiangense

XML Treatment for Amomum carnosum

XML Treatment for Amomum tibeticum

XML Treatment for Lanxangia scarlatina

XML Treatment for Meistera yunnanensis
